# Community participation in the prevention and control of dengue: the *patio limpio* strategy in Mexico

**DOI:** 10.1179/2046904712Z.00000000047

**Published:** 2012-05

**Authors:** Roberto Tapia-Conyer, Jorge Méndez-Galván, Pierre Burciaga-Zúñiga

**Affiliations:** 1 Carlos Slim Health Institute; 2 Hospital Infantil de México Federico Gómez, Ministry of Health; 3 Programa Nacional de Enfermedades Transmitidas por Vectores de México (National Programme for Vector-Borne Diseases), Mexico City, Mexico

**Keywords:** Dengue, Community participation, Integrated vector management, Social mobilisation, Patio limpio

## Abstract

Community participation is vital to prevent and control the spread of dengue in Latin America. Initiatives such as the integrated management strategy for dengue prevention and control (IMS-Dengue) and integrated vector management (IVM) incorporate social mobilisation and behavioural change at the community level as part of a wider strategy to control dengue. These strategies aim to improve the efficacy, cost-effectiveness, environmental impact and sustainability of vector control strategies. Community empowerment is a key aspect of the strategy as it allows the local population to drive eradication of the disease in their environment. Through the *patio limpio* campaign, the concept of community participation has been employed in Mexico to raise awareness of the consequences of dengue. *Patio limpio* consists of training local people to identify, eliminate, monitor and evaluate vector breeding sites systematically in households under their supervision. A community participation programme in Guerrero State found that approximately 54% were clean and free of breeding sites. Households that were not visited and assessed had a 2·4-times higher risk of developing dengue than those that were. However, after a year, only 30% of trained households had a clean backyard. This emphasises the need for a sustainable process to encourage individuals to maintain efforts in keeping their environment free of dengue.

## Background

**Figure 1:**
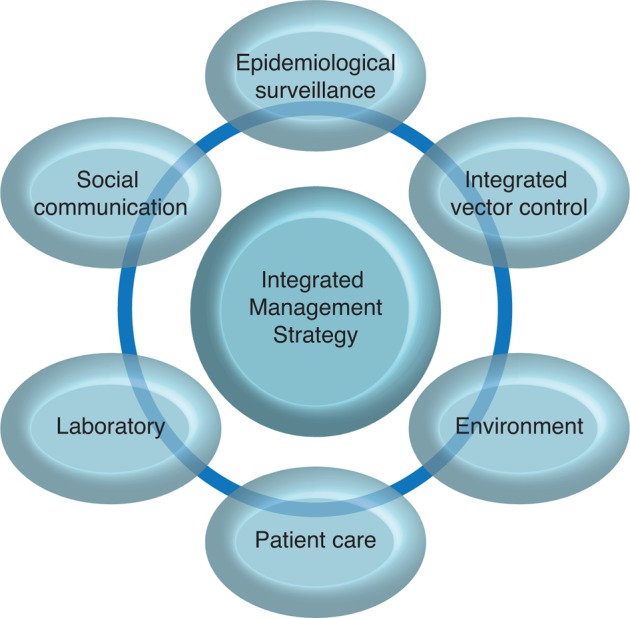
The integrated management strategy (IMS-Dengue) for dengue prevention and control^3^

In order to counter the significant public health burden of dengue in Latin America, the Pan American Health Organization (PAHO) has developed a regional initiative that utilises public participation at community level to encourage behavioural change as part of a wider strategy to control dengue.^1^ The programme, known as the integrated management strategy for dengue prevention and control (IMS-Dengue), aims to promote the integration of key components to prevent and control dengue, including integrated vector management (IVM) (Fig. 1).2,^3^

This article discusses how community participation is vital to prevent and control the spread of dengue in Latin America.

## Integrated Vector Management (IVM)

**Figure 2:**
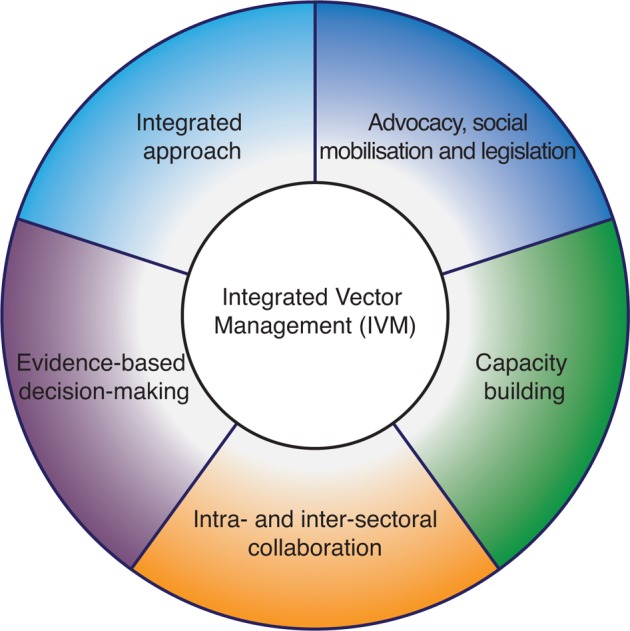
The components of integrated vector management (IVM)^4^

IVM is a global strategic framework first adopted in 2004 by the World Health Organization (WHO) for all vector-borne diseases (Fig. 2).^4^ A further position statement by WHO in 2008 defined IVM as ‘a rational decision-making process for the optimal use of resources for vector control’.^5^ Its key elements are social mobilisation, environmental management, epidemiological and entomological surveillance, and chemical and biological control. IVM policies and strategies aim to improve the efficacy, cost-effectiveness, environmental impact and sustainability of vector control strategies in collaboration with the local community and other public and private sectors. Successful implementation of IVM strategies requires regulatory frameworks, decision-making criteria and execution of procedures that can be implemented down to the lowest administrative level.^5^ This ensures that the concepts of IVM are promoted amongst relevant parties to strengthen legislation and public policy, ensure appropriate pesticide management and empower local communities.^6^

## Social Mobilisation and Communication

Social mobilisation integrates different members of the community, from householders to political leaders, in order to raise awareness of dengue, deliver resources and services and ensure sustained community participation.^7^ The concept of communication for behavioural impact (COMBI) is integral to social mobilisation and should be developed across multiple channels to ensure that information is passed throughout all levels of society.^7^ The initiative aims to affect social behaviour by helping in the planning, implementation and monitoring of communicated actions which promote healthy behaviour. COMBI has been used successfully in other regions.^8^

Community empowerment is one of the most important elements of the IVM strategy, allowing the local population, who most suffer the consequences of dengue, to drive eradication of the disease in their environment.^7^ Public participation is necessary at a number of stages in the local vector control strategy: in assessing the community’s problems and needs, in implementing activities, and in evaluating and monitoring strategy.

Sustainable programmes and modification of individual behaviour are essential in mosquito-control initiatives. This means that individual households must accept responsibility for the control of mosquitoes in their surroundings. However, to maintain sustainability, such efforts should continue as long as the threat of dengue exists and become culturally embedded. To enable this, capacity-building and training of individuals in surveillance, laboratory diagnosis, case management and vector control are important for effective community interventions to be carried out.^7^

As part of the community mobilisation framework, leadership support from local political, religious and community heads is crucial to engage the local population. A multidisciplinary approach, for example between vector control personnel, entomologists, anthropologists, epidemiologists and social marketing experts, is also an important aspect of community mobilisation.^7^

## *Patio Limpio* or ‘clean Backyard’

The concept of community participation as part of social mobilisation has been employed in Mexico in an attempt to raise awareness of the consequences of dengue and to explore how this can impact the risk of contracting the disease.^9^ The initiative also underscores the need for hygiene in the home and instils this into local culture. The strategy, *patio limpio* in Spanish, or ‘clean backyard’, integrates surveillance information and vector control strategies with social mobilisation.

*Patio limpio* consists of training local people to identify and eliminate vector breeding sites in an organised manner. The strategy emphasises the importance of each household in the fight against dengue and the need for all households to work together with the common aim of living in a dengue-free community. The initiative relies on thorough cleaning of the home, which has benefits in addition to the creation of an environment free of dengue. These include an overall clean and presentable home, prevention of bites from other insect types as breeding sites are cleared, and the availability of the yard for recreational family activities. Furthermore, the family can work together as cleaning is an activity that can be carried out by all its members.

The implementation phase of the strategy begins with a local assembly in which the concept of group empowerment and the need for commitment from each household in keeping their surroundings clean and free of breeding sites are explained. Community leaders are identified for each block in the neighbourhood and they are then empowered by being trained and given official identification as ‘block activators’.

The role of block activators is to pass on to families in a given neighbourhood knowledge of how to identify and eliminate breeding sites and help them understand the benefits of keeping the household clean. Families are subsequently responsible for their own environment. Each block activator is responsible for visiting a number of households to train individuals in the process of vector breeding site identification and elimination. Block activators, usually women, also perform a monthly assessment of the area under their supervision and attend community assemblies. Larval indices are shared with block activators and recommendations are provided when expected outcomes are not met, which contributes to the concept of group empowerment.

## Impact of Community Empowerment

The impact of community participation over 1 year (2007) on mosquito-breeding sites in Guerrero, a state in south Mexico, is shown in Table 1.^10^

**Table 1:** Results of community participation on vector breeding sites in Guerrero, Mexico, over a 12-month period (2007)^10^

Number of block activators trained	1192
Average number of households per activator	14·8
Number of backyards visited	5477
Number of clean backyards	2918
Clean backyards index (by recipients)	53·8%
Number of breeding sites identified	19,281

More than 1000 block activators were generated and trained, with an average of approximately 15 households managed by each activator. From a sample of 5477 backyards, approximately 54% (2918) were designated as ‘clean’ and free of breeding sites. Further analysis revealed that households not visited and assessed by a block activator, had a 2·4-times higher risk of developing dengue, compared to those who had been trained and supervised by an activator. In addition, 80% of trained households were able to identify a breeding site and mosquito larvae at the 3-month follow-up visit. However, after a year, only 30% of trained households had a clean backyard and were conscious of the risks associated with breeding sites in their households. This emphasises the need for a sustainable process to encourage individuals to maintain efforts in keeping their environment dengue-free.

## Social Communication in Guerrero

Social communication was an important element in the social mobilisation strategy employed in the State of Guerrero. The communication strategy included displaying 18 signboards and 130 posters, three daily loudspeaker transmissions in areas such as shopping centres and markets throughout the community, and distributing pamphlets to every household visited by block activators. Households already visited and trained were identified by stickers.^10^

## Vaccination and Vector Control in the Community

Even with the potential future availability of a dengue vaccine, community participation is crucial to achieving sustainable control programmes. There is a danger that, with the introduction of a vaccine, community members would regard social mobilisation and community participation as unnecessary. Further education should therefore clarify the role of a vaccine in dengue prevention and how its introduction should be combined with other aspects of vector control, such as community empowerment, in order to maintain preventive practices. The rotavirus vaccine is an example of the need to provide the public with information: communities at risk of infection had to be advised to maintain hygienic practices, despite the existence of a vaccine against rotavirus.^11^

## Conclusion

Undoubtedly, dengue control programmes require a clear, integrated approach with strong community involvement, characteristics that are at the heart of the IVM and social mobilisation concepts. However, for sustained benefit, long-term behavioural modifications at an individual level are imperative. Results of the study carried out in a Mexican community demonstrate the need for community participation programmes to continue as long as dengue continues to be a threat in order to extend the benefits of such initiatives. The lack of continuity of long-term community participation programmes also results in subsequent reluctance by funding and government bodies to invest in and support such initiatives as these strategies fail to achieve desired goals and fulfil expectations. Consequently, these programmes are often relegated to serve as epidemiological projects during dengue outbreaks.

To encourage continuity of programmes, community leaders are effective channels through which to disseminate information, educate communities and catalyse behavioural change at the household level, in turn stimulating progress in the wider community.
